# Secondary Traumatic Stress: Relationship With Symptoms, Exhaustion, and Emotions Among Cemetery Workers

**DOI:** 10.3389/fpsyg.2019.00633

**Published:** 2019-03-22

**Authors:** Lara Colombo, Federica Emanuel, Margherita Zito

**Affiliations:** ^1^Department of Psychology, University of Turin, Turin, Italy; ^2^Department of Philosophy and Education Sciences, University of Turin, Turin, Italy; ^3^Carlo A. Ricciardi Department of Business, Law, Economics and Consumer Behaviour, Università IULM, Milan, Italy

**Keywords:** cemetery workers, secondary traumatic stress, psychological and physical symptoms, psychosocial risks, emotions at work, exhaustion

## Abstract

**Background:** Cemeteries workers are deserving of attention because they are exposed to various psychosocial risks: these workers are subject to painful contacts and daily exposed to a work content linked to death experiences and the emotions associated with them. Secondary trauma develops from this continuous contact with others’ suffering; operators working with this type of traumatic content and dynamic could suffer from emotional disorders (Figley, 1995). Therefore, the secondary traumatic stress (STS) is seen as an occupational risk factor (Bride et al., 2004) and cemetery workers are subject to this risk. Studies on this topic have focused on the operators of emergencies, social, and health sectors; little attention has been given to cemetery workers.

**Aim:** The present study considers the relations between the dimensions composing the STS and the psychological and physical symptoms, the perception of exhaustion, and the positive and negative emotions at work in a group of cemetery workers. Moreover, differences among occupational tasks are explored considering the different possibilities of contact with clients and trauma contagion.

**Methods:** The study included a qualitative phase (interviews and focus groups) and subsequently a quantitative phase (self-report questionnaire) and involved 114 participants in a cemetery organization in northern Italy, divided into technicians employees (TE), technicians and specialists of decoration and garden (TS), gravediggers (GR) administrative and front office employees (AFO). Levels of secondary trauma and psychophysical symptoms were assessed, and correlations were calculated in the total sample and for the different job categories of employees.

**Results:** AFO and TS showed the highest levels of STS and psychophysical symptoms, in particular for symptoms related to anxiety, sadness, insomnia, and gastric and musculoskeletal disorders.

**Conclusion:** This study highlights the importance of considering the STS among also this category of workers, since they are exposed daily not only with death, but also with suffering people; grief and emotional skills are important to cope with these job characteristics cemetery workers are not trained on this. It is important to monitor symptomatic levels not only to avoid chronicity, but also to provide employees with psychological support and training about secondary trauma and its consequences.

## Introduction

The Italian legislation (Legislative Decree no. 81/08) has highlighted the psychosocial and organizational risk factors not only in relation to the safety in the workplace but also to the physical and psychological workers’ wellbeing. Psychosocial risks are defined by the International Labour Organization (ILO) (International Labour Organization [ILO], 1986) as the outcome of multiple factors woven together: the content of the work, the management and organization of work, the conditions of the work environment, the skills, and the needs of workers. Moreover, Cox and Griffiths (1995) also identified psychosocial risk factors connected with social and relational aspects, such as the support of superiors and colleagues, or the organizational climate more generally, that can potentially affect the quality of working life and the perception of safety in the workplace.

Many studies have focused on psychosocial risks in different types of professions, whereas little is known about the specific case of funeral industry operators, and in particular the cemetery service. Then, the funeral industry is considered worthy of investigation in relation to psychosocial risks, both because of its peculiarities and the scarcity of relevant studies in the literature. The theoretical and research contributions on this occupational category are scarce, as are projects for promoting and supporting wellbeing at work (Pinheiro et al., 2012).

From the point of view of working factors, which can lead to feelings of stress and discomfort at work, cemetery services present certain characteristics that are shared with activities carried out by other professions in contact with suffering and death, such as doctors, psychologists, nurses, firefighters, law enforcement officers, emergency operators, etc. (Pinheiro et al., 2012). Unlike the cemetery workers, these categories of personnel have been widely studied in the literature (as shown for example in a meta-analytic study by Cieslak et al., 2014) and are highly recognized and possess social prestige. Workers employed in cemetery services everyday come into contact with the experience of death and the emotions associated with it. These workers can report high levels of suffering at work. Furthermore, they often have to assist with the end of life rites which facilitate the grieving process of their loved ones. Therefore, maybe at higher risk of long terms of psychological distress and physical discomfort, with negative consequences on funeral service quality. The symptoms can include: physical and psychological problems, lack of motivation, dissatisfaction, burnout, absenteeism, counterproductive work behaviors, and addictions development. They may also have negative spillover effects on work–family relations in terms of negative emotions and experiences, and may have significant consequences for the quality of personal and family life (Grzywacz and Marks, 2000).

According to literature, the main psychosocial risks of the funeral work associated with job demands, which can lead to feelings of stress and psychological and physical discomfort, are attributable to the peculiarities of emotional labor (Hochschild, 1983). Emotional labor is characterized by frequent contacts between operators and customers; workers are required to show compassion to the family of the deceased, and to display particular emotions according to the job role. This can lead to experience emotional dissonance, the state perceived by workers when they have to conceal the emotions they actually feel during the relationship with users, in order to conform to the organization requirements (Zapf et al., 2001; Zito et al., 2018). This inserts funerary workers near to those characteristics of the helping professions that have the objective risk of developing negative psychological outcomes, such as compassion fatigue (Figley, 1995) and burnout (Maslach and Leiter, 2000; Argentero and Setti, 2008). Besides, the job also involves skills about grief therapy, not formally provided and for which there is often a total or partial lack of expertise. Grief therapy skills involve the ability to create and maintain an atmosphere suitable for mourning, providing support for funeral arrangements, as well as helping and supporting for the loss.

Moreover, the social image of funeral work is weak, which means there has been little scientific investment in this field in terms of preventive and intervention measures. This weakness is also an additional risk factor for the syndrome of burnout, which can be, in fact, increased by the perception of negative social values of their work (Jourdain and Chênevert, 2010).

In addition to these aspects mentioned above, cemetery workers are subject to the potential development of a secondary or vicarious trauma, based on the assumption that anyone who attends to mourners is constantly exposed to traumatic content and can develop a secondary trauma (Bride et al., 2004). Cemetery workers are constantly in relationship with people affected by traumatic events or exposed to critical and potentially traumatic images/scenes. The cemetery operators, during their work, share the fatigue of trauma, hearing narrative of events, and painful memories for the suffering person (Bride and Figley, 2009). As secondary trauma develops from this continued contact with others’ suffering, operators working with this type of traumatic content and dynamic could experiment emotional disorders (Figley, 1995). Therefore, the secondary trauma is seen as an occupational risk factor (Bride et al., 2004) and cemetery workers are subject to this risk. In fact, the nature of the secondary trauma is empathic: through the vehicle of empathy (on which stay other definitions, such as that of “compassion fatigue”), the others’ experiences are transformed into personal in an assimilative process, so that the effects are felt as if the experience was really a private circumstance. It is no longer just the traumatized person who develops a stress symptomatology, but also those who listen to him/her, who takes care of him/her, and who supports him/her. Therefore, it is not necessary to directly attend the fact, to induce this phenomenon it is sufficient the verbal exposure of painful events. A study by Chrestman (1999) suggests that the secondary trauma shows symptoms similar to the primary trauma, such as intrusive images, avoidance, and distressing or negative emotions. Bride et al. (2004) implemented a measure to detect the secondary trauma, the Secondary Traumatic Stress (STS) Scale, used in this study, since it can capture those symptoms emerging from the exposure to others’ traumatic events. The secondary trauma is composed by three dimensions: first, *intrusion*, referred to thoughts and images of others’ trauma; second, *avoidance*, referred to a general depletion due to an emotional and situational fatigue with others’ pains; third, *arousal*, linked to negative emotions and unpleasant conditions. Since these are symptoms similar to the posttraumatic stress disorder, it could be also a sign linked to functional impairment (Figley, 1995) involving, therefore, also physical problems to the exposure of traumatic events. Moreover, persons exposed to traumatic contents may develop the risk of depression and anxiety symptoms (Bride et al., 2004).

The present study considers the relations between the dimensions composing the STS and the psychological and physical symptoms, the perception of exhaustion, and the positive and negative emotions at work in a group of cemetery employees of a cemetery organization in North Italy. Literature and empirical research on STS is more focused on emergency, social, or medical workers, but little attention is dedicated to cemetery workers that are constantly in contact with death and others’ suffering; moreover, it is difficult to approach and activate project and research in this type of organization. Therefore, this paper detects this professional category contributing to research: the study considers a cemetery organization and explore trauma behaviors and possible links with symptoms and other well-being or distress outcomes. Even if the sample size is small, the study tries to investigate differences among occupational tasks considering, therefore, the different possibility of contact with clients and trauma contagion.

## Materials and Methods

### Ethics Statement

The present study involved human subjects research through self-administered survey. The study was conducted in line with the Helsinki Declaration (World Medical Association, 2001), as well as the data protection regulation of Italy. Even though all study procedures were approved by the Bioethics Committee of the University of Turin (Prot. no. 59372, 23 December 2015), no medical treatments or procedures that could cause social or psychological or pains to participants were considered in the study. The research project was shared with the trade unions and approved by the Board of Directors of the organization. The Department of Psychology and the cemetery organization signed an agreement to ensure anonymity and confidentiality in collecting, analyzing, and publishing data. Participation in the research was voluntary, without receiving any compensation. A cover letter was designed enclosed to the questionnaire to provide an explanation of the participant’s rights to anonymity, voluntary participation, information about the study aims, as well as data treatments. All participants were given instruction to fill out the questionnaire.

### Samples and Procedures

Data were collected using a self-report questionnaire in a cemetery in North Italy. Data analysis proceeded with two phases. The first phase was qualitative and involved both supervisors’ interviews (*N* = 16) and cemetery workers focus group (*N* = 78). Supervisors were interviewed separately from workers, in order to have the associated perception of the work and experiences in general from both the parts. All the workers categories were represented in the interviews and focus groups. In the second phase, a questionnaire was built with the main issues emerged after a content analysis and was administered to all cemetery workers.

As for the questionnaire, participants in the study are 114 (76% of workers) divided into: 32.1% technicians employees (TE – they project and monitor the work that have to be done in the cemetery and guarantee safety, also by being in relation with suffering clients); 25.7% technicians and specialists of decoration and gardens (TS – they meet grieving clients during their work and receive also requests or disapprovals referring to the tombs care); 21.1% gravediggers (GR – they directly face grieving clients during the entombment process or during other procedures such as exhumation); 21.1% administrative and front office employees (AFO – they welcome and guide grieving clients, maintain relations with them, and have to face and solve with their problems linked to bureaucracy). The majority of participants are women (64.3%), with an average age of 48 years (*SD* = 7.66), working on average 38 h per week (*SD* = 9.24), with an average working seniority in the organization of 14 years (*SD* = 9.46).

All study procedures were approved by the Bioethics Committee of the researchers’ University Institution, and the cemetery organization gave the permission to start the research. Researchers administered the questionnaire during dedicated moments in which employees could leave their work and take part to the administration session. Employees signed an informed consent that gave information about the goals of the study, the anonymity of their data, and the voluntary nature of participation in the study. Researchers gave instructions to complete the questionnaire and helped respondents in case of doubts, without influence their answers.

#### Qualitative Phase

Before introducing quantitative measures, it is useful to deepen the qualitative phase. As for the supervisors’ interviews, emerged two main focusing points. The first point is related to the organizational functioning and the management of internal relations. In particular, supervisors reported a growing workers’ distress due to the relational difficulties, linked to the need of understanding how to manage situations with people facing pain. For this reason, within these interviews also emerged the need to make clear all roles in the organization, in order to cope with the different critical work circumstances with efficacy. In fact, supervisors report workers’ insecurity in the management of procedures (even routine procedures) and fear of repercussions in case of errors.

Considering the particular type of job, moreover, supervisors reported the raising need to valorize a complicated work with few acknowledgments and that, indeed, is indicated as a degrading work.

Finally, almost all supervisors report the need of an internal turnover, in particular GR, as a protective factor on health.

The second point emerged from supervisors’ interviews, and is related to the content and the characteristics of the job, linked to the psycho-social risks. In fact, supervisors highlight a high workload and the need of reducing and training about different working issues related to the management of relations with users. Within this circumstance, in particular the AFO area underlines the feeling of being “alone” when facing users’ demands. More in general, all the categories of workers seem to need to strengthen their skills in managing users, mourning, and other people’s emotions. As emerged from supervisors’, in fact, there is an emotional contagion between users and workers, also on the level of health-related concerns (illnesses exalted by contact with the family of the deceased), and this could be linked to the risk of emotional detachment from situations of suffering, that is burnout.

The feeling of being alone, moreover, is related to the need of a professional psychological support to manage grief and pain. This is in particular related to the emotional load caused by the relationship with the families of the deceased and the management of the mourning. Moreover, the emotional load is a source of reported effects of negative spillover in the work–family direction.

As for focus groups, researchers had the possibility to record them and some sentences are therefore reported in the text. In particular, emerged concerns and distress liked to several aspects. One of these is related to the workload, considered the many daily job tasks, and the declared lack of personnel:

*“…the amount of work is so great, things overlap, but not for waste of time on our part, because the operation is really a lot, then the practices are so many”* (AFO).

*“Here everyone in their sectors is overloaded with work, the areas are many, and being few unfortunately we are forced to overload ourselves with work”* (TS).

A crucial point emerged during focus groups refers to the emotional load in the management of mourning and traumatic events: it seems to be a characteristic of all the workers categories, since each of them express difficulties in dealing with and in managing it.

*“Contact with users is difficult. Many parents arrive who have lost their children, and for me, as a parent, I feel very bad. Managing emotion aspects is tiring …”* (TE).

In particular, GR daily face with mourning and contact with death and bodies; the emotional effort still emerges after years of experience. Moreover, GR also have problems in controlling emotions when facing with situations that refer to their personal life or experiences.

“Sometimes tears also fall …and it is a trouble. You cannot be indifferent. Then either old or young does not mean …Unfortunately, we also see children”.

*“Sometimes people say a few phrases to remember the grandmother, the father, the mother …it touches you because maybe it’s something that you lived too, at that moment we become part of that pain too*”.

Moreover, the relation with other’s pain is related to the feeling of emotional dissonance, which does not allow to express the real emotions.

*“I feel like crying …But what should I do? I keep it!”* (TS)

*“This is still a demanding job, being in contact with people, some are crying, some are angry”* (AFO).

Beyond emotional and psychological aspects, it emerges a physical side of the job. In particular, GR and TS feel physic fatigue, describing it as an arduous work.

*“Unfortunately there is the deceased with a light weight, but there is also the deceased heavy and carry it on the shoulder is hard!”* (GR).

*“…so now I sweep all day, which seems like a simple thing, but it’s not …in the end it’s also heavy, you have a backache”* (TS).

Finally, it is observed that some employees show a lived emotional exhaustion, in some cases characterized by cynicism, due to the prolonged exposure to events related to pain and the need to protect themselves:

*“Sometimes we forget to be in a sacred place, but not for a form of little respect, but because after so many years, at the end many things becomes normal, we simply perceive this as a place of work”* (TS).

*“I speak for myself, but it’s a job and it’s done … I looked at it as a job and I did not influence myself. You see them, they cry, they suffer, it’s normal it’s human, you take it like your job, you do it in five minutes, I put myself aside and I immediately leave …this for anyone … and it is quite like a joke …”* (TE).

In some case, employers report to have effect of negative spillover from work to the private life. Workers report that they have experienced stress in some cases compared to the painful situations they daily face.

*“I remember the first times when I came home and I was living badly …I saw the families who live in mourning, who live tragic situations and I was sick”* (AFO).

*“ …I think nobody has ever prepared to manage the pain of others, we are not already able to manage our …”* (GR)

### Measures

In the light of literature and of the qualitative phase and of the different aspects emerged, the questionnaire considered the following measures.

*Secondary traumatic stress* was assessed with 17 items of the Bride et al. (2004) STS Scale. All items were scored on a 5-point scale, ranging from 1 = never to 5 = very often. An example item is “It seemed as if I was reliving the trauma(s) experienced by my client(s).” The scale is comprised of three subscales: first, *intrusion*, referred to thoughts and images of others’ trauma; second, *avoidance*, referred to a general depletion due to an emotional and situational fatigue with others’ pains; third, *arousal*, linked to negative emotions and unpleasant conditions. Scores for the STS general index (all items) and each subscale are obtained by summing the items assigned to each. A confirmatory factor analysis showed a good fit to the data for each subscale: Intrusion [χ^2^(5) = 9.64, *p* = 0.09, RMSEA = 0.09 (0.00, 0.18), CFI = 0.98, TLI = 0.95, SRMR = 0.03], Avoidance [χ^2^(13) = 21.45, *p* = 0.06, RMSEA = 0.07 (0.00, 0.13), CFI = 0.97, TLI = 0.94, SRMR = 0.04], and Arousal [χ^2^(4) = 5.07, *p* = 0.28, RMSEA = 0.05 (0.00, 0.16), CFI = 0.99, TLI = 0.99, SRMR = 0.03]. Cronbach’s alpha for the STS general index in this study was 0.93, 0.82 for Intrusion, 0.82 for Avoidance, and 0.82 for Arousal.

*Psychological and physical symptoms* were measured by eight items of the Avallone and Paplomatas (2005) Multidimensional Organisational Health Questionnaire (MOHQ) scored on a Likert scale from 1 = never to 4 = often. An example item is “Sense of excessive fatigue.” A confirmatory factor analysis showed a good fit to the data: χ^2^(20) = 32.31, *p* = 0.04, RMSEA = 0.07 (0.02, 0.11), CFI = 0.97, TLI = 0.96, and SRMR = 0.05. Cronbach’s alpha was 0.88.

*Exhaustion* was measured by eight items of the Demerouti et al. (2010) Oldenburg Burnout Inventory (OLBI) scored on a Likert scale from 1 = strongly disagree to 4 = strongly agree. An example item is “During my work, I often feel emotionally drained.” A confirmatory factor analysis showed a good fit to the data: χ^2^(12) = 13.65, *p* = 0.32, RMSEA = 0.03 (0.00, 0.11), CFI = 0.99, TLI = 0.99, SRMR = 0.04. Cronbach’s alpha was 0.80.

*Positive emotions at work* was assessed with six items of Warr’s scale (Warr, 1990) scored on a Likert scale from 1 = never to 6 = all of the time. Respondents were asked, thinking of the preceding few weeks, how much of the time their job had made them feel, e.g., “happy” or “calm.” A confirmatory factor analysis showed a good fit to the data: χ^2^(7) = 9.20, *p* = 0.24, RMSEA = 0.05 (0.00, 0.13), CFI = 0.99, TLI = 0.98, SRMR = 0.03. Cronbach’s alpha for the scale in this study was 0.86.

*Negative emotions at work* was assessed with six items of Warr’s scale (Warr, 1990) scored on a Likert scale from 1 = never to 6 = all of the time. Respondents were asked, thinking of the preceding few weeks, how much of the time their job had made them feel, e.g., “depressed” or “gloomy.” A confirmatory factor analysis showed a good fit to the data: χ^2^(8) = 15.58, *p* = 0.05, RMSEA = 0.09 (0.01, 0.16), CFI = 0.97, TLI = 0.94, SRMR = 0.04. Cronbach’s alpha for the scale in this study was 0.84. On the basis of literature, emotions are considered, in this study, as indicators of psychological wellbeing or distress (Diener et al., 1999).

Basic sociodemographic data were collected from all participants: working activity, gender, age, worked hours per week, and organizational seniority.

### Data Analysis

The software IBM SPSS Statistics 24 was used to perform descriptive data analysis, in total sample and in each group separately (TE, TS, GR, and AFO). Moreover, correlations between variables were determined using Pearson’ correlations; non-parametrical correlations among symptoms are calculated with Spearman’s rank correlation coefficient. The psychometric characteristics of scales were examined through a confirmatory factor analysis (maximum-likelihood method of estimation) performed by Mplus 7 (Mutheén and Mutheén, 1998/2012). Cronbach’s alpha coefficient was calculated to test the reliability of each scale. We assessed group differences in the variables’ means using the analysis of variance (*t*-test for independent samples and one-way ANOVA).

## Results

Descriptive statistics allow understanding the general level of STS, the STS sub-dimensions, and the level of physical and psychological symptoms of this sample of cemetery workers. These analyses, in fact, are conducted both on the general sample and within the different groups (Table 1).

**Table 1:** Levels of STS, sub-dimensions, and symptoms in the total sample and within groups: means and standard deviations.

Group	STS	Intrusion	Avoidance	Arousal	Symptoms
Total sample	1.98 (*DS* = 0.98)	2.16 (*DS* = 1.00)	1.90 (*DS* = 0.77)	2.07 (*DS* = 0.91)	2.05 (*DS* = 0.77)
TE	2.01 (*DS* = 0.69)	1.73 (DS = 0.80)	1.73 (*DS* = 0.61)	1.82 (*DS* = 0.64)	2.01 (*DS* = 0.69)
AFO	2.47 (*DS* = 0.87)	2.96 (*DS* = 1.20)	2.16 (*DS* = 1.07)	2.66 (*DS* = 0.98)	2.52 (*DS* = 0.79)
TS	1.73 (*DS* = 0.67)	1.76 (*DS* = 0.78)	1.66 (*DS* = 0.75)	1.79 (*DS* = 0.99)	1.97 (*DS* = 0.69)
GR	1.87 (*DS* = 0.80)	2.03 (*DS* = 0.89)	1.97 (*DS* = 0.89)	1.92 (*DS* = 0.89)	1.67 (*DS* = 0.78)


*STS, secondary traumatic stress (general index); TE, technicians employees; TS, technicians/specialists of decorations and gardens; GR, gravediggers; AFO, administrative/front office.*

Within the total sample, the general level of STS is not severe (*M* = 1.98, *SD* = 0.81, 5-point scale), and looking at the sub-dimensions, the Intrusion is the highest, but under the central point of the scale (*M* = 2.16, *SD* = 1.00), followed by Arousal (*M* = 2.07, *SD* = 0.91) and by Avoidance (*M* = 1.90, *SD* = 0.77). The general level of symptoms within the total sample is placed at the central point of the scale (*M* = 2.05, *SD* = 0.77, 4-point scale).

As for the difference of levels of STS among the different groups, AFO show the highest level (*M* = 2.47, *SD* = 0.87), followed by GR (*M* = 1.87, *SD* = 0.80). The same trend is recurring in the sub-dimensions of the STS, with the highest score for the Intrusion (AFO: *M* = 2.96, *SD* = 1.20; GR: *M* = 2.03, *SD* = 0.89), emerging as the main element of trauma occurring in this sample. The following sub-dimension is Arousal as for AFO (AFO: *M* = 2.66, *SD* = 0.98) but not for GR (*M* = 1.92, *SD* = 0.89), which have a highest score in the Avoidance (GR: *M* = 1.97, *SD* = 0.89), even if after AFO (*M* = 2.16, *SD* = 1.07).

As for psychological and physical symptoms, AFO are again the group with the highest level (*M* = 2.52, *SD* = 0.79), followed by TE (*M* = 2.01, *SD* = 0.69), suggesting that AFO are those who experience more symptoms.

This trend seems to be present also by observing each symptom among groups. Table 2 shows, in fact, the answer percentage in each level of the scale of symptoms: AFO is the category of workers with the highest percentage in high mode (3 or 4). Even if the results of the total sample show low levels (most on mode 1) of symptoms, deepening categories, AFO are those who most experiment all the symptoms, in particular as far as concerns irritability/anxiety (43.5% of responses on mode 4), excessive fatigue (34.8 on mode 4), insomnia (30.4% on mode 4); joint/muscular pain (47.8% on mode 3), and stomachache/gastritis (30.4% on mode 3).

**Table 2:** Symptoms response among the total sample and groups.

		Stomachache/ gastritis	Joint/ muscular pain	Asthma/ respiratory difficulty	Excessive fatigue	Irritability /anxiety	Headache/ concentration difficulty	Insomnia	Depression/ sadness
		
Group	Scale level	% Response	% Response	% Response	% Response	% Response	% Response	% Response	% Response
Total sample	1	42.1	20.2	64.0	30.7	29.8	36.0	45.6	41.2
	2	25.5	13.2	19.3	30.7	31.6	34.2	24.5	29.9
	3	21.1	41.2	7.0	23.7	18.4	20.2	18.4	14.9
	4	11.4	25.4	9.6	14.9	20.2	9.6	11.4	14.0
	*M (DS)*	*2.02 (1.05)*	*2.71 (1.06)*	*1.59 (0.97)*	*2.24 (1.05)*	*2.30 (1.10)*	*2.04 (0.98)*	*1.95 (1.05)*	*2.02 (1.06)*
TE	1	48.6	28.6	74.3	25.7	25.7	28.6	42.9	42.9
	2	22.9	20.0	11.5	40.0	37.2	40.0	20.0	34.3
	3	25.7	37.3	8.6	25.7	20.0	25.7	37.2	14.3
	4	2.9	14.3	5.7	8.6	17.1	5.7	8.6	8.6
	*M (DS)*	*1.83 (0.92)*	*2.36 (1.06)*	*1.45 (0.88)*	*2.18 (0.92)*	*2.29 (1.04)*	*2.09 (0.89)*	*2.03 (1.04)*	*1.89 (0.96)*
AFO	1	21.7	13.0	69.6	8.7	8.7	17.4	26.1	21.7
	2	30.4	4.3	17.4	26.0	30.4	34.7	26.0	30.4
	3	30.4	47.8	4.3	30.4	17.4	26.1	17.4	26.1
	4	17.4	34.8	8.7	34.8	43.5	21.7	30.4	21.7
	*M (DS)*	*2.44 (1.04)*	*3.04 (0.98)*	*1.49 (0.94)*	*2.92 (0.99)*	*2.97 (1.06)*	*2.52 (1.04)*	*2.52 (1.20)*	*2.48 (1.08)*
TS	1	35.7	10.7	53.6	32.1	32.1	39.3	50.0	39.3
	2	32.1	10.7	25.0	28.6	32.1	32.1	35.7	32.1
	3	10.7	46.5	10.7	28.6	17.9	17.9	10.7	14.3
	4	21.4	32.1	10.7	10.7	17.9	10.7	3.6	14.3
	*M (DS)*	*2.18 (1.16)*	*2.99 (0.94)*	*1.73 (1.03)*	*2.20 (1.02)*	*2.24 (1.10)*	*2.00 (1.02)*	*1.67 (0.82)*	*2.04 (1.07)*
GR	1	60.9	26.1	56.5	47.8	47.8	56.5	60.9	60.9
	2	17.3	17.4	26.1	26.0	26.0	30.4	17.3	17.3
	3	17.4	39.1	4.3	13.0	21.7	13.0	17.4	8.7
	4	4.3	17.4	13.0	13.0	4.3	0.0	4.3	13.0
	*M (DS)*	*1.65 (0.94)*	*2.47 (1.08)*	*1.70 (1.05)*	*1.92 (1.09)*	*1.84 (0.94)*	*1.57 (0.73)*	*1.65 (0.93)*	*1.74 (0.11)*


*TE, technicians employees; TS, technicians/specialists of decorations and gardens; GR, gravediggers; AFO, administrative/front office. Scale level: 1, never; 4, often.*

As for TE, they mostly report experimenting joint/muscular pain (37.3% on mode 3), and even if there are highest percentage of answers, also other symptoms are experimented by this category quite frequently, such as insomnia (37.3% on mode 3), stomachache/gastritis (25.7% on mode 3), excessive fatigue (25.7% on mode 3), and headache/concentration difficulty (25.7% on mode 3).

Technicians and specialists of decoration and garden seem to mostly experiment joint/muscular pain (46.5% on mode 3), in line with the characteristic of their job, and within other symptoms that have highest percentage on low scale levels, the most experimented are excessive fatigue (28.6% on mode 3) and stomachache/gastritis (21.4% on mode 4).

Finally, deepening symptoms among GR, they also experiment mostly joint/muscular pain (39.1% on mode 3), according to their job and, among the other symptoms that have most percentage in low levels of the scale, they seem to experiment mostly irritability/anxiety (21.7% on mode 3).

Analysis of variance showed no significant differences among groups. Table 3 shows correlations between the variables in the total sample, Table 4 shows correlations within groups. All the significant correlations between variables were in the expected directions.

**Table 3:** Correlations – STS general index and sub-dimensions with other variables (total sample).

	Secondary traumatic stress	Intrusion	Avoidance	Arousal
1. Age	0.27**	0.32**	0.20*	0.28**
2. Organizational seniority	0.04	0.04	0.03	0.03
3. Symptoms	0.56**	0.52**	0.50**	0.62**
4. Exhaustion	0.38**	0.32**	0.32**	0.44**
5. Positive emotions at work	–0.37**	–0.26**	–0.36**	–0.41**
6. Negative emotions at work	0.43**	0.31**	0.44**	0.49**
7.Stomachache/gastritis	0.23*	0.19*	0.19*	0.29**
8. Joint/muscular pain	0.25**	0.29**	0.22*	0.24**
9. Asthma/respiratory difficulty	0.35**	0.28**	0.40**	0.39**
10. Excessive fatigue	0.46**	0.49**	0.33**	0.51**
11. Irritability/anxiety	0.53**	0.50**	0.45**	0.59**
12. Headache/ concentration difficulty	0.49**	0.36**	0.47**	0.53**
13. Insomnia	0.44**	0.45**	0.35**	0.47**
14. Depression/ sadness	0.55**	0.46**	0.52**	0.59**


*^∗∗^p < 0.01, ^∗^*p* < 0.05.*

**Table 4:** Correlations – STS general index and sub-dimensions with other variables (groups).

	Secondary traumatic stress	Intrusion	Avoidance	Arousal
1. Age	TE = 0.30 AFO = 0.26 TS = 0.23 GR = 0.11	TE = 0.36^∗^ AFO = 0.31 TS = 0.33 GR = 0.09	TE = 0.37^∗^ AFO = 0.05 TS = 0.15 GR = 0.09	TE = 0.36^∗^ AFO = 0.35 TS = 0.18 GR = 0.14
2. Organizational seniority	TE = 0.22 AFO = 0.29 TS = -0.03 GR = 0.04	TE = 0.21 AFO = 0.19 TS = 0.02 GR = 0.08	TE = 0.17 AFO = 0.23 TS = -0.06 GR = 0.03	TE = 0.35^∗^ AFO = 0.35 TS = -0.04 GR = 0.00
3. Symptoms	TE = 0.42^∗^ AFO = 0.58^∗∗^ TS = 0.37 GR = 0.83^∗∗^	TE = 0.45^∗∗^ AFO = 0.47^∗^ TS = 0.30 GR = 0.76^∗∗^	TE = 0.52^∗∗^ AFO = 0.44^∗^ TS = 0.31 GR = 0.85^∗∗^	TE = 0.60^∗∗^ AFO = 0.62^∗∗^ TS = 0.39^∗^ GR = 0.77^∗∗^
4. Exhaustion	TE = 0.23 AFO = 0.57^∗∗^ TS = 0.19 GR = 0.63^∗∗^	TE = 0.15 AFO = 0.60^∗∗^ TS = 0.02 GR = 0.53^∗∗^	TE = 0.28 AFO = 0.28 TS = 0.22 GR = 0.60^∗∗^	TE = 0.31 AFO = 0.62^∗∗^ TS = 0.25 GR = 0.67^∗∗^
5. Positive emotions at work	TE = -0.12 AFO = -0.46^∗∗^ TS = -0.26 GR = -0.62^∗∗^	TE = -0.05 AFO = -0.25 TS = -0.12 GR = -0.50^∗^	TE = -0.11 AFO = -0.46^∗^ TS = -0.29 GR = -0.61^∗∗^	TE = -0.18 AFO = -0.49^∗^ TS = -0.28 GR = -0.65^∗∗^
6. Negative emotions at work	TE = 0.12 AFO = 0.56^∗∗^ TS = 0.40^∗^ GR = 0.66^∗∗^	TE = 0.16 AFO = 0.42^∗∗^ TS = 0.08 GR = 0.57^∗∗^	TE = 0.16 AFO = 0.48^∗∗^ TS = 0.48^∗^ GR = 0.65^∗∗^	TE = 0.28 AFO = 0.57^∗∗^ TS = 0.46^∗^ GR = 0.67^∗∗^
7.Stomachache/gastritis	TE = 0.07 AFO = 0.06 TS = 0.33 GR = 0.33	TE = 0.05 AFO = 0.09 TS = 0.25 GR = 0.30	TE = 0.19 AFO = 0.05 TS = 0.29 GR = 0.31	TE = 0.19 AFO = 0.02 TS = 0.36 GR = 0.34
8. Joint/muscular pain	TE = 0.36^∗∗^ AFO = 0.04 TS = 0.02 GR = 0.48^∗^	TE = 0.49^∗∗^ AFO = 0.19 TS = 0.03 GR = 0.41^∗^	TE = 0.45^∗∗^ AFO = -0.10 TS = -0.04 GR = 0.51^∗^	TE = 0.41^∗^ AFO = 0.01 TS = 0.08 GR = 0.42^∗^
9. Asthma/respiratory difficulty	TE = 0.17 AFO = 0.26 TS = 0.34 GR = 0.80^∗∗^	TE = 0.35^∗^ AFO = 0.06 TS = 0.33 GR = 0.73^∗∗^	TE = 0.35^∗^ AFO = 0.33 TS = 0.24 GR = 0.80^∗∗^	TE = 0.32 AFO = 0.27 TS = 0.38^∗^ GR = 0.74^∗∗^
10. Excessive fatigue	TE = 0.26 AFO = 0.64^∗∗^ TS = 0.18 GR = 0.79^∗∗^	TE = 0.32 AFO = 0.66^∗∗^ TS = 0.13 GR = 0.69^∗∗^	TE = 0.17 AFO = 0.37 TS = 0.14 GR = 0.79^∗∗^	TE = 0.48^∗∗^ AFO = 0.66^∗∗^ TS = 0.22 GR = 0.77^∗∗^
11. Irritability and anxiety	TE = 0.32 AFO = 0.76^∗∗^ TS = 0.36 GR = 0.74^∗∗^	TE = 0.30 AFO = 0.66^∗∗^ TS = 0.38^∗^ GR = 0.63^∗∗^	TE = 0.43^∗∗^ AFO = 0.53^∗∗^ TS = 0.29 GR = 0.75^∗∗^	TE = 0.52^∗∗^ AFO = 0.80^∗∗^ TS = 0.34 GR = 0.72^∗∗^
12. Headache/concentration difficulty	TE = 0.43^∗^ AFO = 0.47^∗^ TS = 0.33 GR = 0.86^∗∗^	TE = 0.31 AFO = 0.19 TS = 0.23 GR = 0.76^∗∗^	TE = 0.45^∗∗^ AFO = 0.53^∗∗^ TS = 0.33 GR = 0.86^∗∗^	TE = 0.51^∗∗^ AFO = 0.53^∗∗^ TS = 0.33 GR = 0.82^∗∗^
13. Insomnia	TE = 0.33 AFO = 0.55^∗∗^ TS = 0.06 GR = 0.60^∗∗^	TE = 0.31 AFO = 0.51^∗^ TS = 0.16 GR = 0.57^∗∗^	TE = 0.42^∗^ AFO = 0.29 TS = 0.04 GR = 0.61^∗∗^	TE = 0.44^∗∗^ AFO = 0.67^∗∗^ TS = -0.02 GR = 0.54^∗∗^
14. Depression and sadness	TE = 0.41^∗^ AFO = 0.65^∗∗^ TS = 0.32 GR = 0.81^∗∗^	TE = 0.43^∗∗^ AFO = 0.46^∗^ TS = 0.16 GR = 0.72^∗∗^	TE = 0.49^∗∗^ AFO = 0.55^∗∗^ TS = 0.32 GR = 0.86^∗∗^	TE = 0.53^∗∗^ AFO = 0.71^∗∗^ TS = 0.37 GR = 0.73^∗∗^


*TE, technicians employees; TS, technicians/specialists of decorations and gardens; GR, gravediggers; AFO, administrative/front office; ^∗∗^*p* < 0.01, ^∗^*p* < 0.05.*

In the total sample (Table 3), both STS general index and sub-dimensions were strongly and positively correlated with symptoms (*r*_STS_ = 0.56, *r*_INTR_ = 0.52, *r*_AV OID_ = 0.50, *r*_AROU_ = 0.62) and exhaustion (*r*_STS_ = 0.38, *r*_INTR_ = 0.32, *r*_AV OID_ = 0.32, *r*_AROU_ = 0.44) and negative emotions (*r*_STS_ = 0.43, *r*_INTR_ = 0.31, *r*_AV OID_ = 0.44, *r*_AROU_ = 0.49), negatively correlated with positive emotions at work (*r*_STS_ = -0.37, *r*_INTR_ = -0.26, *r*_AV OID_ = -0.36, *r*_AROU_ = -0.41). With regard to psychological and physical symptoms, in total sample both STS general index and sub-dimensions show significant correlations with all symptoms, in particular with psychological symptoms as depression/sadness (*r*_STS_ = 0.55, *r*_INTR_ = 0.46, *r*_AV OID_ = 0.52, *r*_AROU_ = 0.59), irritability/anxiety (*r*_STS_ = 0.53, *r*_INTR_ = 0.50, *r*_AV OID_ = 0.45, *r*_AROU_ = 0.59), headache/concentration difficulty (*r*_STS_ = 0.49, *r*_INTR_ = 0.36, *r*_AV OID_ = 0.47, *r*_AROU_ = 0.53), excessive fatigue (*r*_STS_ = 0.46, *r*_INTR_ = 0.49, *r*_AV OID_ = 0.33, *r*_AROU_ = 0.51), and insomnia (*r*_STS_ = 0.44, *r*_INTR_ = 0.45, *r*_AV OID_ = 0.35, *r*_AROU_ = 0.47). Correlations show that age has a positive relationship with STS general index and also with Intrusion, Avoidance, and Arousal (*r*_STS_ = 0.27, *r*_INTR_ = 0.32, *r*_AV OID_ = 0.20, *r*_AROU_ = 0.28). Age and organizational seniority presents both a positive correlation with joint/muscular pain, mainly *r* = 0.39 and *r* = 0.34. Non-parametrical correlations between symptoms show a stronger relationship between symptoms, in particular between physical and psychological symptoms within them.

In TE group (Table 4), both STS general index and sub-dimensions present no significant correlations with exhaustion and positive and negative emotions. STS general index and Intrusion, Avoidance, and Arousal sub-dimensions are positively correlated with symptoms (*r*_STS_ = 0.42, *r*_INTR_ = 0.45, *r*_AV OID_ = 0.52, *r*_AROU_ = 0.60), joint/muscular pain (*r*_STS_ = 0.36, *r*_INTR_ = 0.49, *r*_AV OID_ = 0.45, *r*_AROU_ = 0.41), and depression/sadness (*r*_STS_ = 0.41, *r*_INTR_ = 0.43, *r*_AV OID_ = 0.49, *r*_AROU_ = 0.53). Furthermore, STS general index is also correlated with headache/concentration difficulty (*r* = 0.43); Intrusion is also correlated with asthma/respiratory difficulty (*r* = 0.35); Avoidance is also correlated with asthma/respiratory difficulty (*r* = 0.35), irritability/anxiety (*r* = 0.43), headache/concentration difficulty (*r* = 0.45), and insomnia (*r* = 0.42); and Arousal is also correlated with excessive fatigue (*r* = 0.48), irritability/anxiety (*r* = 0.52), headache/concentration difficulty (*r* = 0.51), and insomnia (*r* = 0.44).

In AFO group (Table 4), STS general index and Intrusion, Avoidance, and Arousal sub-dimensions were correlated with outcomes, in particular exhaustion (*r*_STS_ = 0.57, *r*_INTR_ = 0.60, *r*_AROU_ = 0.62, Avoidance not significant) and negative emotions (*r*_STS_ = 0.56, *r*_INTR_ = 0.42, *r*_AV OID_ = 0.48, *r*_AROU_ = 0.57), negatively correlated with positive emotions at work (*r*_STS_ = -0.46, *r*_AV OID_ = -0.46, *r*_AROU_ = -0.49, Intrusion not significant). STS general index and sub-dimensions are strongly and positively correlated with symptoms (*r*_STS_ = 0.58, *r*_INTR_ = 0.47, *r*_AV OID_ = 0.44, *r*_AROU_ = 0.62), irritability/anxiety (*r*_STS_ = 0.76, *r*_INTR_ = 0.66, *r*_AV OID_ = 0.53, *r*_AROU_ = 0.80), and depression/sadness (*r*_STS_ = 0.65, *r*_INTR_ = 0.46, *r*_AV OID_ = 0.55, *r*_AROU_ = 0.71). In particular, STS general index is correlated too with excessive fatigue (*r* = 0.64), irritability/anxiety (*r* = 0.76), and headache/concentration difficulty (*r* = 0.47); Intrusion is also correlated with excessive fatigue (*r* = 0.66) and insomnia (*r* = 0.51); Avoidance is also correlated with headache/concentration difficulty (*r* = 0.53); and Arousal is also correlated with excessive fatigue (*r* = 0.66), headache/concentration difficulty (*r* = 0.53), and insomnia (*r* = 0.67).

In TS group (Table 4), significant correlations among STS general index, Intrusion, Avoidance, and Arousal sub-dimensions and symptoms are scant. STS general index is positively correlated with negative emotions at work (*r* = 0.40). Intrusion is correlated with irritability/anxiety (*r* = 0.38); Avoidance is correlated with negative emotions (*r* = 0.48); and Arousal is correlated with symptoms (*r* = 0.49), negative emotions (*r* = 0.46), and asthma/respiratory difficulty (*r* = 0.38).

In GR group (Table 4), both STS general index and sub-dimensions were strongly and positively correlated with symptoms (*r*_STS_ = 0.83, *r*_INTR_ = 0.76, *r*_AV OID_ = 0.85, *r*_AROU_ = 0.77) and exhaustion (*r*_STS_ = 0.63, *r*_INTR_ = 0.53, *r*_AV OID_ = 0.60, *r*_AROU_ = 0.67) and negative emotions (*r*_STS_ = 0.66, *r*_INTR_ = 0.57, *r*_AV OID_ = 0.65, *r*_AROU_ = 0.67), negatively correlated with positive emotions at work (*r*_STS_ = -0.62, *r*_INTR_ = -0.50, *r*_AV OID_ = -0.61, *r*_AROU_ = -0.65). STS general index and Intrusion, Avoidance, and Arousal sub-dimensions are positively and strong correlated with all symptoms, except from stomachache/gastritis, in detail with joint/muscular pain (*r*_STS_ = 0.48, *r*_INTR_ = 0.41, *r*_AV OID_ = 0.51, *r*_AROU_ = 0.42), asthma/respiratory difficulty (*r*_STS_ = 0.80, *r*_INTR_ = 0.73, *r*_AV OID_ = 0.80, *r*_AROU_ = 0.074), excessive fatigue (*r*_STS_ = 0.79, *r*_INTR_ = 0.69, *r*_AV OID_ = 0.79, *r*_AROU_ = 0.77), irritability/anxiety (*r*_STS_ = 0.74, *r*_INTR_ = 0.63, *r*_AV OID_ = 0.75, *r*_AROU_ = 0.72), headache/concentration difficulty (*r*_STS_ = 0.86, *r*_INTR_ = 0.76, *r*_AV OID_ = 0.86, *r*_AROU_ = 0.82), insomnia (*r*_STS_ = 0.60, *r*_INTR_ = 0.57, *r*_AV OID_ = 0.61, *r*_AROU_ = 0.54), and depression/sadness (*r*_STS_ = 0.81, *r*_INTR_ = 0.72, *r*_AV OID_ = 0.86, *r*_AROU_ = 0.73).

## Discussion

The aim of the study was to explore the relations between STS and symptoms, and between STS and well-being and distress indicators, such as positive emotions at work, negative emotions at work and exhaustion. In particular, the study focused both on the general level of symptoms and STS, and on the specific dimensions composing STS and single symptoms among both the total sample of cemetery workers and the specific workers’ category.

Although this is an exploratory study, results highlight critical points that have to be deepened in the light of literature and of the practical implications.

In particular, the first qualitative phase allowed to identify critical points linked to the management of emotions in general and to others’ emotions, a topic related to the STS. Employers, in fact, express difficulties in the experience of others’ mourning both for the situations in which they should help user, and in situations in which they shift others’ pain to personal experience. This side could particularly be linked to the experience of exhaustion which can deplete physical resources, but also emotions. As for the physical aspect, it has to be underlined the fatigue that some workers face in doing their work, an aspect detected in this study with the general aim to endorse what is declared, but also to understand possible exhaustion effects on physical distress.

In particular, results highlighted that AFO is the weakest category, since it shows the highest level of STS and symptoms in general, also within correlations analysis.

In the total sample, the general level of STS appears under the central point of the scale, indicating even a presence of the secondary traumatization, in particular as for the intrusion dimension, in line with literature suggesting this element as a peculiar trauma symptom and a characteristic of STS (Bride et al., 2004). In fact, the intrusion dimension is the one with the highest level also among the different category of workers. As mentioned, AFOs are the category that particularly is subject to STS, showing general level of STS reaching the central point of the scale and beyond, as intrusion and arousal. The dimension of intrusion is also remarkable among the GR category, consistently with the characteristics of the job. Indeed, these particular categories of workers that have to daily face with a suffering client is subject to the continuous contact with customer asking for help, and support, which calls emotional dissonance (Zapf et al., 2001; Zito et al., 2018), but requires also emotional and grief skills. This leads the operators in constant contact with others’ suffering experiences, activating the process of secondary trauma as reaction to others’ emotional demands (Jenkins and Baird, 2002).

The level of symptoms follows the same trend of the STS level, in the total sample: the general level is beyond the central point of the scale, suggesting a distributed level of distress among cemetery workers. Again, AFOs are the category with the highest level of symptoms, together with TS.

Deepening the specific symptoms, administrative, and front office workers in particular seem to experiment more frequently irritability/anxiety, excessive fatigue, insomnia, joint/muscular pain, stomachache/gastritis, but also depression/sadness. It is interesting to note that also other TEs are subject to the same trend, in particular as for insomnia and joint/muscular pain, symptoms peculiar of GR, and TS, considering the particular physical effort they are required to do. All symptoms are in line with a trauma framework. In fact, anxiety sensitivity and depression are associated with symptoms experimented by trauma and secondary trauma exposed individual (Bride et al., 2004; Mahaffey et al., 2017), but also gastrointestinal, musculoskeletal, and other generalized psychical discomfort are related to a traumatization (Parcella et al., 2013; Milligan-Saville et al., 2017).

Correlations between symptoms and STS general level and sub-dimensions highlight again a critical situation for the two categories that are more in contact with suffering clients: AFOs and GR, showing high correlations. Moreover, this category of workers shows highest correlations also between STS and STS dimensions and exhaustion and positive and negative emotions, operationalized as indicators of emotional wellbeing and discomfort at work. Detecting these aspects is important, since it identifies both the emotional disruption as a risk factor for cemetery operators, and the experimentation of secondary traumatization, also in order to precisely understand if the individual is subject to secondary trauma or other physical or psychological pain, such as burnout (Bride et al., 2004). In this light, it is important also to monitor the relation and the frequency of symptoms, in order to avoid that they become chronic (Milligan-Saville et al., 2017), undermining the quality of life. This is functional and important also to protect workers from the risk of somatization of emotional and traumatization experiences. In particular, anxiety and respiratory symptoms can have the effect of confusing the person living a discomfort event, conducting the person to develop a psychological distress which can be experienced as dangerous symptoms (Mahaffey et al., 2017), near to a form of hypochondria. Indeed, this could explain also the fact that AFOs and GR experiment highest level of STS and symptoms: being constantly in contact with suffering clients that they help in bureaucracy and technical processes, knowing all the elements of death explained by the family of the defunct, they somatized their diseases. This element emerged during the qualitative phase of the research and it is very useful to explain these results: workers in contact with the death and others’ death stories often move others’ pain to the own, appropriating of their symptoms. This is in line with the empathic nature of STS and the identification and consequent intrusion of images and thoughts (Bride et al., 2004), and with the cumulative nature of traumatization, which increase with age (Brewin et al., 2000; Haug et al., 2004). Although in this study age show significant correlation with STS in total sample, seniority does not show significant correlation with STS.

### Limitations and Future Studies

The limitations of this study are the use of a small sample and of a unique organizational context. This not allows generalization of the results, and the use of a self-report questionnaire and a cross-sectional research design that does not establish of sure relations of causality between variables. However, this study permits to identify symptoms and relations with STS among a particular worker category, and also among the different tasks category. This could allow even inhibit risk factors among cemetery workers, by monitoring the level of possible STS and symptoms, and activating preventing and training program. To deepen these aspects, future longitudinal studies should consider the effect of STS also on private life, in order to detect spillover effects and to identify hypochondria or dysfunctional behaviors associated to trauma or work stress. Studies on this issue should also consider multigroup analysis to better capture differences among group and within groups, in order also to understand the symptomatology dynamics according to the type of job tasks. Moreover, future studies should collect additional data in order to perform more complex analyses to understand the presence of causal effects within variables. For this reason, Structural Equations Models should be performed, also to design studies considering the influence of mediation or moderation variables. Multigroup analysis should therefore use this type of analyses. Finally, future studies should detect the posttraumatic growth (Tedeschi and Calhoun, 2004), an unexplored issue that should be functional to postulate positive interventions against the effect of STS, in the light of positive psychology, avoiding the depletion of emotional resources and of somatizations, with consequences on health (Lahav et al., 2016).

## Conclusion and Practical Implications

This study highlights the importance of considering the secondary trauma among also this category of workers, since they are constantly in contact not only with death, but also with suffering people that are in turn living a recent pain and that are often asking for grief and emotional skills that cemetery workers are not trained to give (Pinheiro et al., 2012). Workers should receive focused training on these processes in order to manage others’ pain, but also their emotions. Being aware of the STS characteristics and have the possibility to have a support on their personal experience could be a very useful instrument to protect against physical symptoms and traumatization in general. Organizational culture, indeed, has a key role on the trauma experience (Cieslak et al., 2014) and one of the main instruments to face this is the continuous support for stress management (Figley and Kleber, 1995; Jenkins and Baird, 2002). Social support, in fact, are depicted as crucial to contrast negative effects (Cohen and Wills, 1985) and a specific training should be offered also to supervisors in order to recognize and manage traumatization through intervention and practical or emotional support. Among implications, other strategies helping the managing of the risk of traumatization are related to supervision of traumatized operators and also training on self-care strategies (Bride et al., 2004). In fact, in this regard, a useful implication is also related to the periodic supervision, socialization to the role for new hires, and possible shifts to allow employees to recover in relation to certain activities which expose more than others to the management of death.

Moreover, cemetery workers should receive specifically training in order to promote the development of personal resources: this allow employee to face traumatic situations and could be a crucial point for the person and the quality of the life in general, and for the organization in programming focused intervention that not only adjusts what is damaged, but also empowers and activates positive circles at work.

Finally, it is important to consider the perceived workload that could depend also by both physical fatigue, and the management of emotions. Beyond the number of workers provided for a specific job, it could be a wellbeing strategy to project internal turnover, in order to decrease disease in general and the perceived emotional loads and exhaustion. In particular, the emotional load caused by the relationship with the families of the deceased and the management of the mourning can lead to the risk of burnout related to those workers declaring emotional detachment.

## Data Availability

The datasets generated for this study are available on request to the corresponding author.

## Author Contributions

LC, FE, and MZ made a contribution to the present study, designed the research and collected the data, and wrote the manuscript. MZ and FE carried out data analysis and interpretation. The final version of the manuscript has been approved for submission by all authors; they are accountable for the whole work.

## Conflict of Interest Statement

The authors declare that the research was conducted in the absence of any commercial or financial relationships that could be construed as a potential conflict of interest. The handling Editor declared a shared affiliation, though no other collaboration, with the author LC at the time of review.
